# Case of Aneurism of the Arch of the Aorta; Intense Dyspnœa; Absence of Recurrent Laryngeal Paralysis; Peculiar Difficulties in Diagnosis; Death; Autopsy

**Published:** 1883-11

**Authors:** Frank Dudley Beane

**Affiliations:** New York City


### Clinical Reports.

### Case of Aneurism of the Arch of the Aorta; Intense Dyspnœa; Absence of Recurrent Laryngeal Paralysis; Peculiar Difficulties in Diagnosis; Death; Autopsy.

By Frank Dudley Beane, A. M., M. D.,
of New York City.

The following case would seem to be of special interest in a diagnostic and pathological point of view. It came under my observation during October, 1882.

H. M. P., mulatto, æt. 42 years, Connecticut, married. Had the usual diseases of childhood, with no sequelæ. At the age of eighteen years he commenced to earn his livelihood, and, at the same time, to imbibe of all kinds of alcoholic liquors to excess. This habit was continued till his twenty-seventh year, when, on marrying, he decreased his alcoholic libations to about one-third of the former quantity. Since then the amount taken daily has fluctuated between three and eight "drinks" daily. Has been addicted to excessive smoking since the age of sixteen.

In his twenty-fourth year he contracted what his shipmates told him was the "initial lesion" (no physician's opinion to be obtained, being upon the ocean on a long voyage at the time), which he cauterized with stick nitrate, and promptly healed. It, however, reopened two months later, remained unhealed for five or six months, when an eruption appeared upon the whole body, particularly localized about the joints. Character of eruption: Round blotches, red base, surmounted by white scales, on lifting which a dark red, elevated spot, of the size of a small lentil, appeared; discrete over the surface of the body, confluent at the joints. Intense itching.

On the appearance of this eruption he placed himself under the care of a regular physician of this city, who treated him for "secondary," although the patient had at no time (then or since)

adenopathy, buccal, pharyngeal, scalp, or any of the symptoms of syphilis, unless the eruption be so considered.

At the end of six months' treatment the eruption had disappeared, leaving pigmented spots, which did not disappear for four or five years. Patient continued taking this physician's medicine over a period of two years.

Family history good, excepting that of his brother, who was believed to have died from cancer of the groin, which penetrated to the deeper structures, causing death from hemorrhage; his mother died from uterine cancer.

*Present Disease:* Patient enjoyed health till about two years ago, when, as steward of a steamship plying between this port and Aspinwall, he was exposed to great wet and dampness during day and night, and his duties were laborious. Severe nasal catarrh first appeared, followed by bronchial catarrh, without dyspnœa. Cough, expectoration and pectoral distress fluctuated till the spring of 1881, when these symptoms began to improve, and during that summer he was nearly, although not wholly, free from them. There had been no loss of flesh, and only an occasional night-sweat.

In the following fall and winter the above symptoms became worse, a little ameliorated in the spring and summer again, but not as much so as the same season of the previous year.

In June, 1882, a peculiar dyspnœa was added to the other symptoms, being confined to inspiration. Constant in character, it was marked by paroxysms of severity in the morning and evening. It became especially aggravated during the first week in November. Since the appearance of this symptom, the patient could not assume the recumbent position, so perfectly terrible the dyspnœa became on attempting to do so. He slept in his chair, his head resting upon his arm placed upon the table in front of him. From three to five hours' sleep during the twenty-four was all he could obtain.

Curiously enough, up to the time he came under my observation, his general health had become but very little deteriorated, his strength, appetite, etc., being good. Indeed, he did not abandon work, as a waiter, till five weeks previous (i. e., about Sept. 1, 1882).

*Physical Examination:* (Laryngoscopic)—Intense congestion of the mucous membrane of the epiglottis and larynx. No laryngeal ulcer, tumor, or stricture. (Auscultation and percussion)—Examination of the lungs was negative, except the sign of incomplete expansion of the right lung, and dry, harsh respiratory murmur throughout both lungs.

Cardiac apex beat strong, and about two and a half inches below and a little to the outside of a line drawn vertically through the nipple. Heart sounds normal, barring a certain amount of faintness. Dullness extended from the second intercostal space to about two inches below and a little outside of the nipple on the outer line; from the same level and right edge of the sternum to the same apex on the inner line; the upper line commenced at the right edge of the sternum, and was traced to nearly a point cut by a vertical line upward from the nipple. Over the sternum, on a level with the lower edge of the first rib downward for two inches, was a circumscribed area of absolute flatness.

Having made repeated stethoscopic examinations, I was able but once to detect a rough, rasping bruit over the normal location of the aortic arch.

No signs of diaphragmatic trouble, or of disease of the liver, spleen, or kidneys, could be found till a short time before his death, when albuminuria (mild) set in.

Although I was perfectly satisfied with my opinion, that the patient had never suffered from syphilis, and leaned greatly toward the diagnosis of aortic aneurism (more particularly after I had detected the above-mentioned bruit), I determined to give him (after a short trial of small doses of potassium iodide, strychnia, and quebracho) large doses of potassium iodide and minute doses of mercury, on the ground of giving him the benefit of the (serious) doubt regarding syphilis. I reasoned that, whether syphilis or aneurism, the iodide should benefit him.

Accordingly, on Nov. 13, 1882, he began upon hydrarg. biniodid., gr. j; potassii iodid., ʒvj; ammon. chlorid., ʒiij; tr. opii camph., *f*℥ss; aqua font., *f*℥iij; syr. simp., ad *f*℥vj. M. Sig: Dessertspoonful, diluted, after each meal. On Dec. 5th, I increased the potassium iodide to ℥jss in the above prescription, these doses being continued daily for a couple of weeks more. At times during this treatment (which included almost constant and complete rest) the dyspnœa was somewhat improved, but not markedly so. After about six weeks' trial it was abandoned. Strychnia, in doses of gr. one-twelfth four times a day, gave no relief. Quebracho, even in large doses, was inert in this case. Infusion of digitalis, given to tolerance, gave little or no result.

Having candidly stated to the patient my inability to differentiate positively between aortic aneurism, cancerous mediastinal growth, and tracheal stricture, although I favored the diagnosis of aneurism, and informed him of the very serious nature of his condition, I advised him to see Drs. Geo. M. Lefferts and Clinton Wagner, to each of whom I sent a brief account of the patient's case, with my views thereon. Those gentlemen very carefully examined the patient (Dr. Wagner several times). Dr. Lefferts wrote me that his "diagnosis * * * is mediastinal tumor (aneurism of the arch) compressing trachea (I cannot see point of stenosis with the laryngoscope)." Dr. Wagner wrote: "I think the dyspnœa is caused by pressure from an intra-thoracic tumor, probably mediastinal. I do not think there is an aneurism of the aorta. An aneurism of the size sufficient to produce dyspnœa such as he has would surely press upon the recurrents. He would then have paralysis of one or both vocal cords. His larynx and trachea are normal. I could detect no bruit." In a later communication he said: "I am sure we shall find a mediastinal tumor of some kind."

Let me here state that I had fully weighed the absence of signs of pressure upon the recurrents, and had favored the diagnosis of aortic aneurism despite the lack of this very important sign for three reasons: First, absence of syphilitic (according to my view) and traumatic history led me to eliminate tracheal stricture; second, absence of great loss of flesh and strength, of peculiar color of skin and countenance, and of adenopathy, militated seriously against cancer of mediastinum; third, I had undoubtedly heard a bruit over the arch.

The patient struggled heroically with his disease, which rapidly increased about the first of February of the present year, till death closed the scene and released him from terrible suffering (despite all justifiable means of palliation) on February 19th.

Post mortem held on the 20th by Drs. G. Fairfax Whiting and —— Simpson, in my presence, disclosed a complete displacement of the heart downward and to the left, the normal space occupied by that organ being replaced by an apparently solid tumor, which, at the point corresponding to absolute dullness, was very firmly adherent to the sternum. It was also pretty firmly adherent to the surrounding tissues. There were evidences of old pleuritic adhesions. There was a peculiar circumscribed infiltration of the apex of the left lung of the size of a large English walnut. Dr. Whiting removed the heart and intimately attached tumor and the lungs for careful examination at home, and his subsequent report was as follows: "After taking the specimen home and dissecting the supposed tumor from its attachments to the heart and lungs, it was found to be an aneurism of the aorta. The entire cavity of the aneurism was filled up with laminated fibrine, leaving only the natural calibre of the artery open for the passage of the blood current, which condition accounts for there being no bruit present. There was considerable concentric hypertrophy of the walls of the left ventricle."

The autopsy was confined to the chest, the family being unwilling to allow a more complete one.

Believing this to have been an unique case of aortic aneurism of large size, and especially interesting structure, without the least sign of pressure upon the recurrent laryngeal nerves, I herewith submit it to the readers of the JOURNAL without further comment.

